# Immunomodulatory properties of umbilical cord mesenchymal stromal cells in immune disorders: immunoregulatory functions, therapeutic progress, and engineering strategies for enhanced efficacy

**DOI:** 10.3389/fimmu.2026.1948742

**Published:** 2026-09-15

**Authors:** Chenglian Xie, Yongliang Lu, Jie Ma, Manjun Deng, Chuanchuan Liu

**Affiliations:** Laboratory of Stem Cells and Regenerative Medicine, Qinghai University Affiliated Hospital, Xining, Qinghai, China

**Keywords:** autoimmune diseases, cell therapy, clinical translation, immune cells, immunomodulation, umbilical cord−derived mesenchymal stromal cells

## Abstract

Immune disorders remain a major global health challenge, as conventional immunosuppressive therapies are often hampered by severe side effects and unsatisfactory long-term efficacy. Umbilical cord-derived mesenchymal stromal cells (UC-MSCs) have emerged as a promising therapeutic modality for various immune disorders and balance immune homeostasis by regulating various immune cells and secreting anti-inflammatory mediators. We systematically review and synthesize preclinical and clinical evidence supporting UC-MSC treatment for major immune diseases, with graft-versus-host disease, systemic lupus erythematosus, inflammatory bowel disease, and rheumatoid arthritis being prime examples. Despite the promising therapeutic outcomes, therapeutic efficacy and large-scale clinical manufacturing of UC-MSC therapies are constrained by multiple challenges. This review also systematically examines the major translational bottlenecks for UC-MSCs in treating immune disorders, including cellular heterogeneity, non-uniform preparation standards, impaired homing and biodistribution, and insufficient long-term safety data. Finally, two main avenues for enhancing UC-MSC therapeutic potential are summarized, including optimized culture strategies, such as hypoxic preconditioning, cytokine priming, and three-dimensional spheroid culture, and genetic engineering. It is hoped that we will be able to gain a deeper understanding of the development of stable and scalable UC-MSC interventions for immune diseases.

## 1 Introduction

Inflammation is a double-edged sword. While acute inflammation serves as a protective host response, chronic and dysregulated inflammation contributes to the pathogenesis of numerous diseases, including autoimmune disorders, metabolic syndrome, graft-versus-host disease (GVHD), neurodegenerative conditions, and infectious diseases such as coronavirus disease 2019 (COVID-19) (1–4). Autoimmune diseases, a major category of immune dysregulation, affect approximately 5%–8% of the global population, with females accounting for roughly two-thirds of cases (5). When broader immune-mediated inflammatory diseases (e.g., asthma, atopic dermatitis, psoriasis) are included, the prevalence of immune dysregulation-related conditions rises to about 10% worldwide (6).

Current immunomodulatory strategies, including corticosteroids, non-steroidal anti-inflammatory drugs, disease-modifying antirheumatic drugs, and biologic agents targeting specific cytokines (e.g., tumor necrosis factor-alpha (TNF-α) inhibitors and interleukin-6 (IL-6) receptor blockers), have revolutionized the management of inflammatory diseases (7). However, these approaches have prominent drawbacks, including generalized immunosuppression that elevates susceptibility to infection and progressive loss of therapeutic efficacy resulting from immunogenicity and disease progression (8, 9). These shortcomings underscore the need for novel therapeutic platforms that combine immunomodulation with regenerative capacity.

Mesenchymal stromal cells (MSCs) have emerged as a promising cell-based platform with potent immunomodulatory properties. Initially recognized for their differentiation potential, MSCs are now appreciated primarily for their paracrine-mediated immunomodulatory functions (10). Unlike conventional immunosuppressants, MSCs respond to the inflammatory microenvironment, exerting context-dependent immune regulation—suppressing excessive inflammation while preserving host defense capacity. Furthermore, MSCs secrete a broad array of trophic factors that promote tissue repair, offering a dual benefit of immunomodulation and regeneration (11).

While MSCs can be isolated from various tissues including bone marrow and adipose tissue, umbilical cord-derived MSCs (UC-MSCs) offer distinct advantages. Umbilical cord tissue is routinely discarded after delivery, enabling non-invasive cell collection with no reported adverse complications for donors (12). These cells also feature a youthful cellular phenotype, marked by faster proliferation, longer telomere length, and stronger multilineage differentiation capacity relative to adult tissue-derived MSCs (13). Moreover, UC-MSCs exert potent immunoregulatory effects via abundant secretion of immunomodulatory mediators including indoleamine 2,3-dioxygenase (IDO), human leukocyte antigen-G (HLA-G), and prostaglandin E2 (PGE_2_), alongside low immunogenicity driven by weak major histocompatibility complex class II (MHC class II) expression; this property allows off-the-shelf allogeneic infusion with no requirement for HLA matching (14, 15). In addition, each umbilical cord can generate large quantities of MSCs, supporting standardized cell banking and large-scale industrial manufacturing (16).

This review aims to provide a comprehensive and up-to-date overview of UC-MSC immunomodulation. We begin by dissecting the immunomodulatory mechanisms of UC-MSCs (Section 2). Then, we summarize preclinical/translational research evidence covering major categories of immune-mediated disorders, along with advances in relevant clinical trials (Section 3). Section 4 systematically discusses core obstacles and unresolved issues restricting the clinical translation of UC-MSC immunomodulatory therapies. Finally, we address emerging strategies to improve UC-MSC immunomodulatory potency, including various preconditioning protocols and genetic engineering in Section 5. **Figure 1** illustrates the core content of this manuscript.

**Figure 1:**
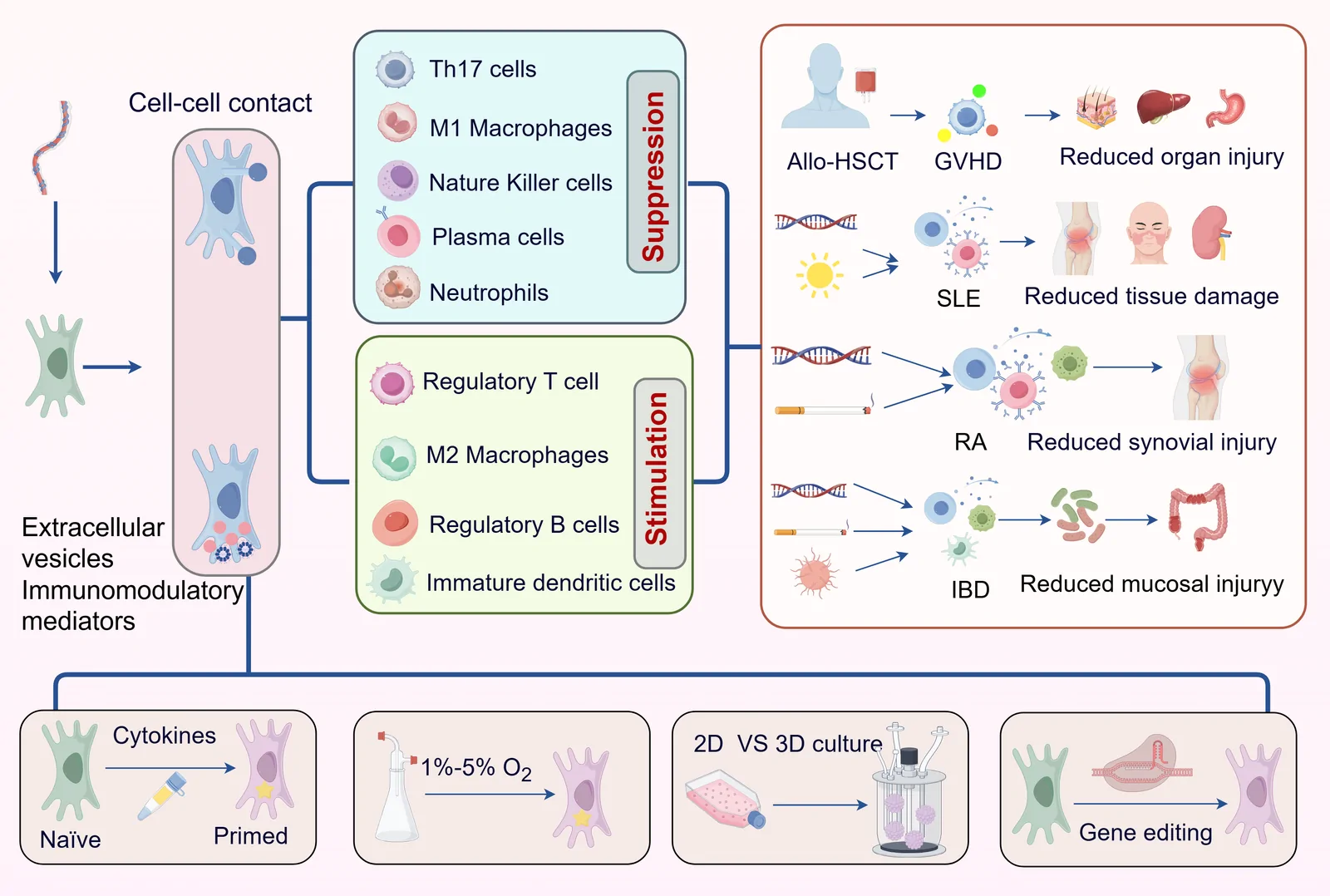
Graphical abstract. Umbilical cord-derived mesenchymal stem cells (UC-MSCs) are isolated from Wharton’s jelly of umbilical cords, a tissue rich in diverse bioactive components. UC-MSCs regulate virtually all types of immune cells through direct cell-cell contact and various soluble factors. Their immunomodulatory functions can be broadly categorized into two aspects: immunosuppression, which inhibits Th17 cells, M1 macrophages, NK cells, plasma cells, and neutrophils; and the induction of reparative and regulatory immune populations, including Tregs, Bregs, immature DCs (imDCs), and M2 macrophages. Through these coordinated mechanisms, UC-MSCs exert therapeutic effects in immune-mediated disorders such as graft-versus-host disease (GVHD), systemic lupus erythematosus (SLE), rheumatoid arthritis (RA), and inflammatory bowel disease (IBD). This immunomodulatory potency may be further enhanced by strategies including cytokine preconditioning, hypoxic culture, three-dimensional culture, and genetic engineering.

## 2 Immunomodulatory mechanisms of UC-MSCs

The immunomodulatory effects of UC-MSCs are mediated through a complex and interconnected network. A key mechanism involves the direct reprogramming of immune cell phenotypes and functions through inflammatory signaling modulation and soluble mediator secretion (17–19). In this section, we categorize the evidence according to the core mechanisms of UC-MSC action, as illustrated in **Figure 2**. UC-MSCs construct this regulatory network through two complementary modes. The first is long-range regulation via soluble cytokines and UC-MSC-derived extracellular vesicles (UC-MSC-EVs). The second is short-range regulation through direct cell-to-cell contact. These mechanisms act in concert to trigger downstream signaling cascades that modulate the functional phenotypes of diverse immune cell subsets.

**Figure 2:**
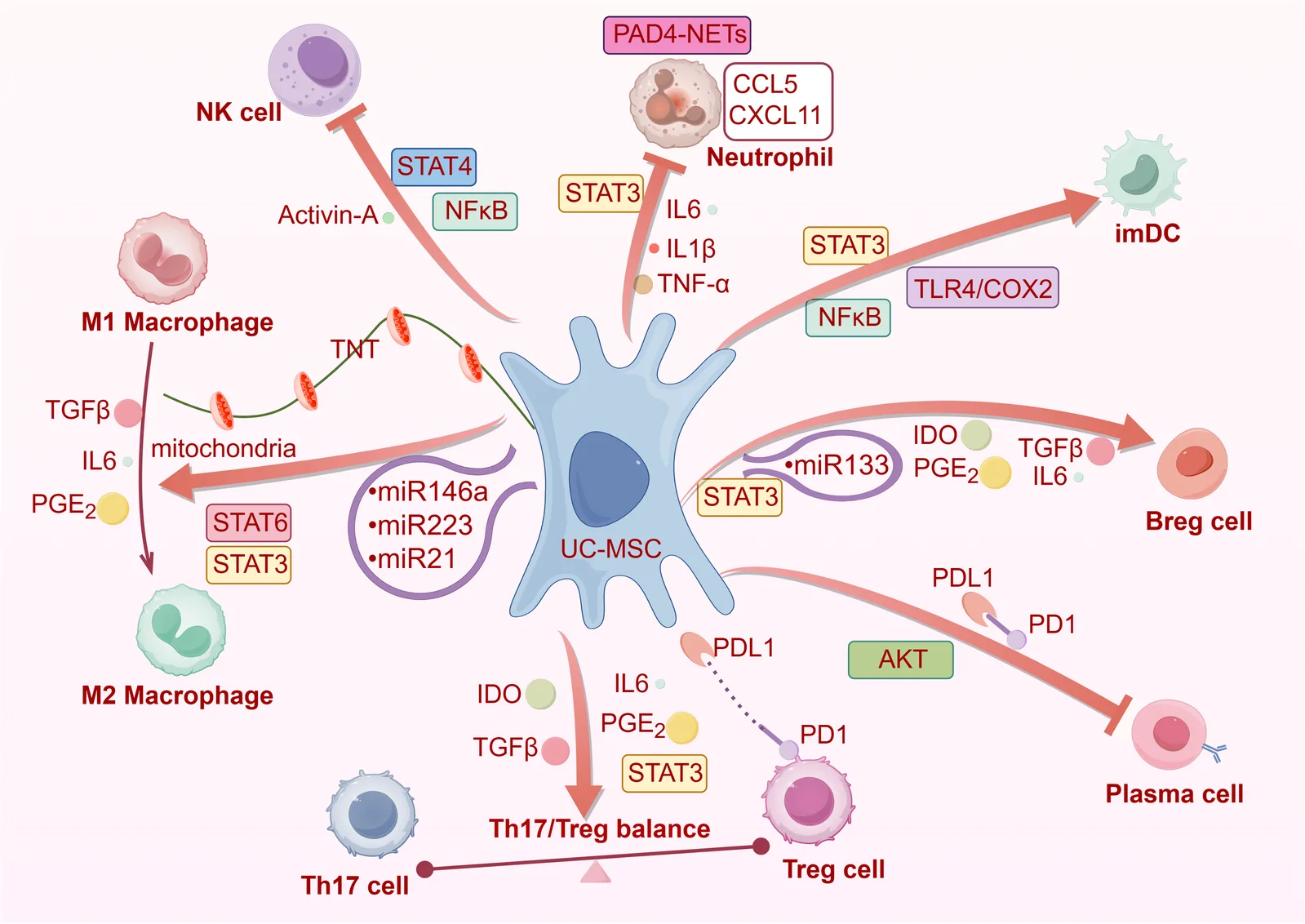
Immunomodulatory mechanisms of UC-MSCs. UC-MSCs modulate immune responses through three major mechanisms: soluble factors (e.g., TGF-β, PGE_2_, IDO), cell-free particulate mediators, including miRNA-containing extracellular vesicles and tunneling nanotubes—termed TNTs—that mediate mitochondrial transfer to macrophages and promote M2 polarization, and direct cell-cell contact via membrane-bound molecules, such as PD-L1. Functionally, UC-MSCs suppress T-cell proliferation, rebalance the Th17/Treg-cell equilibrium, dampen NK-cell cytotoxicity and plasma-cell activation, inhibit neutrophil activity, and block DC maturation while maintaining an immature state; they also promote Treg and Breg generation, collectively reshaping the immune network. These effects are mediated through JAK/STAT, NF-κB, and phosphoinositide 3-kinase (PI3K)/AKT signaling, leading to the resolution of excessive inflammation, reduction of tissue injury, and restoration of immune homeostasis.

### 2.1 UC-MSCs modulate immune cells through soluble mediators

UC-MSCs secrete a broad range of soluble mediators, jointly establishing a sophisticated immunomodulatory network that targets both innate and adaptive immune cells, suppressing excessive inflammation and promoting immune tolerance.

Transforming growth factor beta (TGF-β) is a highly pleiotropic signaling molecule. On the one hand, it synergizes with PGE_2_ and IDO to suppress lymphocyte activation and proliferation, thereby restricting the effector functions of CD4^+^ and CD8^+^ T cells (20–22). On the other hand, TGF-β alone drives the differentiation of naive CD4^+^ T cells into regulatory T cells (Tregs) (23, 24). Additionally, in combination with IL-6 and PGE_2_, TGF-β activates signal transducer and activator of transcription 3 (STAT3) and STAT6 signaling to promote M2 macrophage polarization, and these cells display upregulated expression of CD163, CD206, arginase-1, and IL-10 (25, 26). In the humoral immune compartment, TGF-β cooperates with IL-6, IDO, and PGE_2_ to inhibit the terminal differentiation of B cells into plasma cells and promote the generation of regulatory B cells (Bregs) (27–29).

Beyond lymphocyte-oriented immunomodulation, UC-MSCs also shape innate immune responses, with prominent regulatory effects on neutrophils. In a zebrafish model of bacterial pneumonia, UC-MSCs reduce neutrophil recruitment and suppress the production of proinflammatory mediators, including interleukin-1 beta (IL-1β), IL-6, and TNF-α (30). Furthermore, in traumatic brain injury-related acute lung injury models, UC-MSCs block neutrophil extracellular trap formation and pulmonary neutrophil sequestration by suppressing peptidyl arginine deiminase 4 activity (31).

### 2.2 UC-MSCs suppress immune responses through cell-to-cell contact

Beyond soluble paracrine factors, UC-MSCs also execute immunosuppressive activities through membrane-bound molecules in a cell-contact-dependent manner. Programmed death-ligand 1 (PD-L1) is a key surface molecule that mediates such contact-based regulation. Mechanistically, PD-L1 engagement on UC-MSCs promotes the differentiation of naive CD4^+^ T cells into Tregs and inhibits the terminal differentiation of B cells into plasmablasts and plasma cells (23, 24, 27).

The regulatory crosstalk between UC-MSCs and natural killer (NK) cells is context-dependent and confers dual immunomodulatory properties. In most experimental settings, UC-MSCs exert inhibitory effects on NK cells by suppressing the activation of the cytotoxic CD56^dim^ NK subset through downregulation of TNF-α and perforin expression (32). Additionally, NK cells co-cultured with UC-MSCs downregulated natural killer group 2 D expression and exhibited reduced levels of CD107a, perforin, and granzyme B (33). However, when primed with hypoxia (2% O_2_) combined with proinflammatory stimuli (IL-1β, TNF-α, interferon gamma (IFN-γ)), UC-MSCs markedly attenuate NK cell-mediated cytotoxic responses (34). Under certain culture conditions, UC-MSCs can even act as feeder cells to support the ex vivo expansion of NK cells, an effect that occurs in culture systems supplemented with exogenous cytokines including IL-2, IL-15, and Fms-like tyrosine kinase 3 ligand (35). These diverse regulatory effects reflect the functional plasticity of UC-MSCs. Their bidirectional modulation of NK cell activity indicates that the immunoregulatory functions of UC-MSCs are conditional rather than universal, requiring careful consideration of both experimental conditions and the local microenvironment in future studies.

### 2.3 UC-MSCs transmit regulatory signals through extracellular vesicles

UC-MSC-EVs, which carry microRNAs (miRNAs) as their core cargo, constitute a third mode of intercellular communication that functions independently of soluble factors and cell–cell contact. At the level of lymphocytes, UC-MSC-EVs reshape the Th17/Treg balance by modulating the IL-6/STAT3/IL-17 axis and induce Breg generation via miR-133 (36, 37). In myeloid cells, EV-derived miR-146a, miR-21, and miR-223 inhibit nuclear factor kappa-B (NF-κB) signaling, repressing proinflammatory M1 polarization while driving anti-inflammatory M2 reprogramming of macrophages (38). Additionally, mitochondrial transfer via tunneling nanotubes (TNTs) remodels macrophage bioenergetics toward oxidative phosphorylation to metabolically sustain the M2 phenotype (39). Beyond miRNAs, UC-MSC-EVs also carry a variety of other bioactive molecules, including proteins, lipids, and metabolites, which act in concert to fine-tune immune responses (40). These findings underscore the capacity of UC-MSC-EVs to orchestrate multilineage immune regulation through coordinated modulation of intracellular signaling and direct functional reprogramming of immune cells, positioning them as a versatile and promising cell-free therapeutic platform.

### 2.4 Convergence on intracellular signaling pathways and restoration of immune homeostasis

Although the three signal delivery modes, namely, soluble mediators, contact-dependent signaling, and EV-mediated cargo delivery, differ in their upstream routes, they converge upon common downstream signaling networks in recipient cells. Such molecular convergence allows UC-MSCs to orchestrate coordinated immune responses across diverse immune lineages through a finite set of intracellular signaling hubs.

UC-MSCs exert immunomodulatory functions through multiple intracellular signaling cascades. The Janus kinase (JAK)/STAT axis represents one key signaling integration hub. STAT3 drives Treg differentiation, inhibits Th17-cell development, and promotes Breg generation, while STAT3 and STAT6 jointly facilitate M2-type macrophage polarization (25, 28, 37). Through STAT3 repression, UC-MSCs also limit neutrophil chemotaxis and infiltration by reducing keratinocyte-derived secretion of C-C motif chemokine ligand 5 (CCL5 and C-X-C motif chemokine ligand 11 (CXCL11) (41). In NK cells, UC-MSC-secreted Activin-A exerts targeted inhibitory effects by reducing STAT4 and NF-κB phosphorylation, attenuating IL-12/IL-18-driven IFN-γ production (42). In addition, AKT (also known as protein kinase B) and p38 signaling mediate the UC-MSC-induced suppression of B-cell proliferation and antibody secretion (38, 43).

In dendritic cells (DCs), UC-MSCs downregulate Toll-like receptor 4 and NF-κB-p65 expression, which is associated with reduced nuclear NF-κB-driven transcription of pro-inflammatory genes, resulting in decreased pro-inflammatory cytokine production and enhanced IL-10 secretion. Collectively, these changes drive the acquisition of a tolerogenic DC phenotype (44). DCs exposed to UC-MSCs display diminished antigen-presenting capacity and downregulated maturation markers, and consequently fail to efficiently activate naive T cells (44, 45). In addition, UC-MSCs downregulate retinoid-related orphan receptor-γt (RORγt) and upregulate forkhead box P3 (FOXP3), thereby suppressing Th17-cell differentiation and promoting Treg differentiation to shift the Th17/Treg balance (46, 47). Collectively, by integrating multiple intracellular signaling pathways, UC-MSCs remodel both adaptive and innate immune dysregulation and restore immune homeostasis, conferring protective effects against multiple immune-mediated disorders, including GVHD, rheumatoid arthritis (RA), systemic lupus erythematosus (SLE), and inflammatory bowel disease (IBD) (22, 48–52).

Importantly, all of the above immunomodulatory effects are context-dependent; the regulatory direction of UC-MSCs, whether enhancement or suppression, relies on the local inflammatory milieu, metabolic state, and activation status of target cells. In a murine asthma model, UC-MSCs unexpectedly attenuated M2 polarization and reduced IL-12 and TNF-α expression, illustrating that even canonical pathways can yield divergent outcomes under different pathological conditions (53). Similarly, the modulation of chimeric antigen receptor (CAR) T cells by UC-MSCs exhibits bidirectional effects depending on the tumor microenvironment (50). Under high tumor-burden conditions, UC-MSCs boost the antitumor capacity of CD19 CART cells by inducing Th17-biased differentiation of CD8^+^ NK-like cytotoxic T lymphocytes and relieve cytokine release syndrome via inhibition of hyper-activated macrophages (54). Conversely, UC-MSCs can improve CART-cell persistence and function via mitochondrial transfer, while suppressing CART-cell cytotoxicity through the IDO-1 and stanniocalcin-1 pathways; both facets of this bidirectional regulation have been reported in the existing literature (55, 56).

UC-MSCs exert potent immunomodulatory effects. However, most current evidence derives from co-culture systems or discrete animal models, which limits extrapolation of these findings to human disease settings.

## 3 Major disease categories and clinical trial progress

The broad immunomodulatory effects of UC-MSCs lay the mechanistic foundation for their therapeutic potential. A growing body of preclinical and clinical research has investigated UC-MSC-based interventions across multiple disease contexts. The following section focuses specifically on four representative immune-mediated diseases, namely, GVHD, SLE, RA, and IBD, examining their therapeutic efficacy and underlying mechanisms. Where applicable, evidence obtained from other MSC sources is also briefly highlighted to provide insights for advancing UC-MSC-based therapy. Relevant studies are summarized in **Table 1**, which outlines study design, cell-delivery protocols, sample size, experimental outcomes, and key findings.

**Table 1:** Summary of clinical and preclinical studies on UC-MSCs for major immune disorders.

Major disease	Ref	Study design/model	Route/dose	n	Outcome	Key findings
GVHD	(57)	Preclinical: Murine model (C57BL/6 and BALB/c)	IV, 5 × 10^5^ cells/mouse	/	Survival improved; Clinical score reduced; Organ pathology alleviated	UC-MSCs alleviate murine aGVHD via lymphocyte apoptosis
	(58)	Preclinical: Murine model (C57BL/6 and BALB/c)	IV,/	/	GVHD incidence and severity reduced	MDSC enrichment in target organs; CXCL1-CXCR2 axis- mediated mechanism
	(59)	Preclinical: Murine model (C57BL/6 and BALB/c)	IV, 5 × 10^5^ cells/mouse	9–15	UC-MSC: 60% survival (vs. 0% in aGVHD); Clinical score 4 (vs. 8); Histopathology scores reduced in lung, liver, colon, small intestine; IFN-γ, TNF-α, ST2 reduced	PHS suppresses Th1 and alleviates aGVHD via PPAR-γ/NF-κB/STAT4/T-bet
	(60)	Preclinical: Murine xenogeneic model (NSG mice)	IV, 5 × 10^5^ + IP, 1 × 10^6^ cells/mouse	48–96	Best group: 100% survival; CD8^+^ T cells reduced; weight maintained; clinical score minimized	Syngeneic UC-MSCs + UCB-MNCs at high dose best inhibit GVHD, governed by matching and timing
	(61)	Preclinical: Murine xenogeneic model (NSG mice)	IV,/	/	Three weekly injections of primed UC-MSCs improved survival, reduced histological GVHD scores in the liver and skin	IFN-γ-primed GMP UC-MSCs alleviate preclinical GVHD and benefit refractory GVHD treatment
	(62)	Case series	IV,/	8	aGVHD improvement; Neutrophil/platelet recovery	Safe; effective against aGVHD; enhances hematopoiesis
	(63)	Cohort study	IV, 1 × 10^6^ cells/kg	86	28-day ORR 52.3%; 100-day OS 43.7%; Long-term OS 11.6% (108 months)	Worse response with intestinal + liver aGVHD; No severe AEs
	(64)	Multicenter RCT	IV, 1 × 10^6^ cells/kg	192	aGVHD III–IV: 2.1% vs. 21.9%; cGVHD: 27.6% vs. 45.5%; severe cGVHD: 5.5% vs. 14.8%; 3-year GRFS: 62.4% vs. 32.0%	OS comparable; UC-MSC prophylaxis reduced GVHD and improved GRFS without compromising survival
SLE	(65)	Preclinical: MRL/lpr mice	IV, 5 × 10^5^ cells/mouse	20	UC-MSC: 4-week efficacy; renal pathology improvement; UC-MSC + IL-2 no added benefit	UC-MSCs outperform IL-2 in sustained Treg elevation and renal protection
	(37)	Preclinical: MRL/lpr mice	IV,/	38	Proteinuria, serum creatinine, renal pathology, splenic index, anti-dsDNA IgG all reduced; Survival improved	UC-MSC-EVs modulate splenic/renal T-cell subsets and renal STAT3/IL-17A signaling
	(51)	*In vitro* study	/	27	SLE and HVs show reduced BR3/CD40/PD-1; SLE has higher PGE_2_; HV shows elevated CD80	UC-MSC secretome modulates B-cell surface markers, exerting distinct effects in SLE patients and HVs
	(66)	Phase I/II clinical trial	IV, 1 × 10^6^ –5 × 10^6^ cells/kg	58	Primary: safety (day 1–28); Secondary: SLEDAI-2K ≥4 reduction at week 24	Ongoing trial; results pending
	(67)	RCT (study protocol)	IV, 1 × 10^6^ cells/kg	96	Primary: renal response rate at 6 and 12 months	Trial protocol; results pending
	(68)	Case series (Phase I, ongoing)	SC, 6 × 10^7^ cells	10	Safety, tolerability; SLEDAI-2K, BILAG, QoL	Ongoing trial; results pending
	(69)	Case series (interim)	SC, 6 × 10^7^ cells	2	SLEDAI-2K: 20→6 (Pt1, day 168); 10→0 (Pt2, day 84); Prednisone: 25→0 mg/day	Safe, only transient minor AEs; sustained anti-inflammatory effect
	(70)	Case series	IV, 1 × 10^6^ cells/kg	22	SLE patients with reduced apoptotic PBMCs, increased plasma PGEM post-transplant	UC-MSC uptake of ACs amplifies NF-κB/COX2/PGE_2_-mediated T suppression; SLE patients gain higher plasma PGEM with fewer apoptotic monocytes post-transplant
RA	(71)	Preclinical: CIA rat model	IV, 5 × 10^5^ cells/mouse	30	En-MSCsC/I eased joint injury and inflammation, matched MSC efficacy, with better joint tropism and less lung trapping.	En-MSCsC/I modulated synovial/myeloid cells, blocked CXCL12-CXCR4, drove regenerative FLS, and repaired cartilage via SEMA3E.
	(72)	Preclinical: CIA rat model	IV, 1 × 10^6^ cells/mouse	60	Reduced joint swelling/erythema, cartilage loss, and inflammatory infiltration; induced synovial apoptosis	IL-1β -induced LFA-1/ICAM-1 adhesion and TRAIL/DR4/5 caspase-dependent apoptosis; UC-MSCs vanished in joints by day 20.
	(73)	Preclinical: CIA rat model	IV, 5 × 10^5^ cells/mouse	25	Relieved arthritis, reduced infiltration/cartilage injury, elevated Tregs, and suppressed CD8^+^ T/NK activation	UC-MSC plus mutant IL-2 yielded stronger Treg expansion than a single treatment.
	(74)	Cohort study	IV, 40 × 10^6^ cells/patient	64	1/3-year follow-up: normal blood, liver/kidney, and immunoglobulin indices; reduced ESR, CRP, RF, anti-CCP, DAS28, and HAQ	UC-MSC plus DMARDs exerted 3-year safe and sustained efficacy in RA patients.
IBD	(75)	Preclinical: DSS-induced colitis mouse model	IV, 2 × 10^6^ cells/mouse	24	Reduced DAI, colon length restored, lower TNF-α/IFN-γ/IL-4/IL-17, higher TGF-β1/IL-10 and Tregs; CD126^lo^ showed no improvement	CD126^hi^ UC-MSCs secrete TGF-β1 to rebalance Treg cells and alleviate inflammation
	(17)	Preclinical: DSS-induced colitis mouse model	IV, 1 × 10^6^ cells/mouse	27	Relieved DUOX2-related oxidative stress via T-cell regulation	Suppress DUOX2 oxidative stress via T-cell immunity regulation
	(76)	Preclinical: TNBS-induced colitis rat model	IV, 1 × 10^6^ cells/mouse	60	Elevated survival and body weight, decreased DAI, CMDI, and histopathological scores, alleviated colonic congestion, edema, and ulceration	UC-MSCs alleviated TNBS-induced colitis in rats, supporting their therapeutic potential for CD.
	(77)	Preclinical: DSS-induced colitis mouse model	IP, 1 × 10^6^ cells/mouse	18	Attenuated colitis manifestations; UC-MSC homing to epithelium and lamina propria, lowered ER stress levels, elevated Xbp1 splicing.	UC-MSCs alleviate DSS colitis by modulating epithelial ER stress and Xbp1 splicing, showing therapeutic prospect in ulcerative colitis.
	(76)	Case series	Local Subm, 60 × 10^6^ cells/patient; IV, 1 × 10^6^ cells/kg next day	17	Lower SES-CD, CDAI, CRP, IL-8, calprotectin; higher Hb; 47% endoscopic response, 17.65% mucosal healing, no severe adverse events	Combined local and IV UC-MSCs safely improve refractory CD clinically and endoscopically
	(18)	Case series	IV, 1 × 10^6^ cells/kg	41	73.2% response, 41.5% remission at 2 months; decreased Mayo, endoscopic and pro-inflammatory cytokine levels, higher IL-10, Hb, Alb; age -independent factor (OR = 0.875), no severe AEs	UC-MSCs are safe and effective for ulcerative colitis; age impairs efficacy via lowered progerin and inflammatory cytokines
	(17)	Case series	IV, 1 × 10^6^ cells/kg	26	2/6m response 80.8%/75.0%, remission 46.2%/37.5%, mucosal repair in responders	UC-MSCs restore mucosal immunity, preserve gut barrier, and yield ulcerative colitis clinical remission

UC-MSC, umbilical cord mesenchymal stromal cell; GVHD, graft-versus-host disease; SLE, systemic lupus erythematosus; RA, rheumatoid arthritis; IBD, inflammatory bowel disease; IV, intravenous injection; SC, subcutaneous injection; Subm, submucosal injection; IP, intraperitoneal injection; OS, overall survival; MDSC, myeloid-derived suppressor cells; CXCL, C-X-C motif chemokine ligand; CXCR, C-X-C motif chemokine receptor; PHS, phytosphingosine; ST2, suppression of tumorigenicity 2; PPAR-γ, peroxisome proliferator-activated receptor gamma; NF-κB, nuclear factor kappa-light-chain-enhancer of activated B cells; STAT, signal transducer and activator of transcription; T-bet, T-box transcription factor TBX21; UCB-MNCs, umbilical cord blood mononuclear cells; NSG, NOD scid gamma; GMP, good manufacturing practice; OR, odds ratio; ORR, overall response rate; AEs, adverse events; RCT, randomized controlled trial; GRFS, graft-versus-host disease-free, relapse-free survival; HV, healthy volunteer; SLEDAI-2K, Systemic Lupus Erythematosus Disease Activity Index-2000; QoL, quality of life; BILAG, British Isles Lupus Assessment Group index; Pt, patient; ACs, apoptotic cells; COX2, cyclooxygenase-2; PGE_2_, prostaglandin E2; PGEM, prostaglandin E metabolite; CIA, collagen-induced arthritis; FLS, fibroblast-like synoviocytes; LFA-1, lymphocyte function-associated antigen-1; ICAM-1, intercellular adhesion molecule-1; TRAIL, tumor necrosis factor-related apoptosis-inducing ligand; DR4, death receptor 4; Tregs, regulatory T cells; DMARDs, disease-modifying antirheumatic drugs; HAQ, Health Assessment Questionnaire; ESR, erythrocyte sedimentation rate; CRP, C-reactive protein; RF, rheumatoid factor; anti-CCP, anti-cyclic citrullinated peptide antibody; DAS28, Disease Activity Score 28; BR3, BLyS receptor 3; PBMC, peripheral blood mononuclear cell; En-MSCsC/I, enucleated C-C chemokine receptor 2/IFN-γ-overexpressing UC-MSCs; SEMA3E, semaphorin 3E; DAI, Disease Activity Index; TNF-α, tumor necrosis factor-α; IFN-γ, interferon-γ; TGF-β1, transforming growth factor-β1; DUOX2, dual oxidase 2; TNBS, trinitrobenzenesulfonic acid; CMDI, Colonic Mucosal Damage Index; CD, Crohn’s disease; DSS, dextran sulfate sodium; ER, endoplasmic reticulum; XBP1, X-box binding protein 1; SES-CD, Simple Endoscopic Score for Crohn’s Disease; CDAI, Crohn’s Disease Activity Index; Hb, hemoglobin; Alb, albumin.

### 3.1 GVHD

GVHD is a severe alloreactive complication arising from donor lymphocytes infiltrating and damaging host organs post allogeneic hematopoietic stem cell or bone marrow transplantation (78). UC-MSCs exert potent immunosuppressive activity to alleviate established acute GVHD (aGVHD) and facilitate hematopoietic reconstitution, representing a promising cellular therapeutic strategy for the prophylaxis and treatment of GVHD.

#### 3.1.1 Preclinical/translational research

The therapeutic efficacy of UC-MSCs in GVHD has been extensively validated in preclinical animal models. In a murine aGVHD model, co-transplantation with UC-MSCs significantly improved survival and alleviated target organ pathology, accompanied by increased CD4^+^ T-cell apoptosis and reduced serum IFN-γ and TNF-α levels (57). Mechanistically, this protection has been attributed to multiple pathways. A study showed that UC-MSCs enriched myeloid-derived suppressor cells in target organs through CXCL1 secretion, as revealed by RNA sequencing (58). A distinct metabolic mechanism was reported by another group, who found that UC-MSC infusion elevated serum phytosphingosine, which suppressed Th1 differentiation and reduced IFN-γ and TNF-α expression in CD4^+^ T cells (59).

Hansen et al. systematically compared 16 treatment regimens varying in UC-MSC dose, culture expansion, timing, and genetic matching, identifying the simultaneous infusion of culture-expanded syngeneic UC-MSCs at 1 × 10^6^ cells as the most effective strategy, which achieved 100% survival and preserved weight and clinical scores. Cox analysis confirmed syngeneic matching and simultaneous administration as the key determinants of efficacy (60). Notably, more recent efforts have moved beyond natural efficacy toward optimized strategies. IFN-γ preconditioning of UC-MSCs significantly enhanced survival and histological outcomes in a xenograft model, with the benefit mediated by IDO activity (61). Collectively, these preclinical findings establish UC-MSCs as a promising cell-based therapy for GVHD and provide mechanistic rationales for further refinement.

#### 3.1.2 Clinical trial progress

Peng first reported the clinical use of UC-MSCs for ameliorating aGVHD in 2010. Three patients received therapeutic UC-MSC infusions for steroid-refractory aGVHD, whereas another five received prophylactic UC-MSC infusions co-administered with cord blood hematopoietic transplantation. UC-MSC intervention relieved aGVHD symptoms, accelerated neutrophil and platelet reconstitution, and was well tolerated without adverse reactions. The authors suggest that UC-MSCs offer a safe and effective alternative cell source for GVHD therapy (62). In a cohort of 86 patients suffering from severe steroid-refractory aGVHD (grade IV disease accounting for 82.6%), UC-MSC infusion yielded a 52.3% overall response rate (ORR) at day 28. The corresponding 100-day and long-term survival rates reached 43.7% and 11.6%, respectively. Patients with combined intestinal and hepatic aGVHD exhibited significantly worse therapeutic responses, whereas no serious adverse events were documented throughout treatment (63).

Prevention of GVHD is a primary objective when treating patients with allogeneic hematopoietic cell transplantation (HCT). A multicenter, open-label randomized controlled trial demonstrated that prophylactic UC-MSC infusion markedly lowered the incidence of both acute and chronic GVHD after haploidentical HCT. This protective effect was most prominent against severe aGVHD, whereas overall survival was comparable across the two arms. This finding indicates that the prophylactic use of UC-MSCs against GVHD does not compromise patient survival. Importantly, the nearly twofold increase in GVHD-free, relapse-free survival within the UC-MSC group highlights the promising value of UC-MSCs as a prophylactic regimen for optimizing long-term post-transplant prognosis (64).

The administration strategies established by other MSC products offer a transferable framework for UC-MSC trial design. Dose-exploration data for bone marrow-derived MSCs (BM-MSCs) in GVHD show a common single-dose range of 1–2 × 10^6^/kg, and infusion protocols have evolved from single injection toward repeated-dose regimens (79, 80). However, simply raising the infusion frequency does not necessarily yield improved therapeutic efficacy, and optimal dosing intervals remain to be further investigated (81).

### 3.2 SLE

SLE is a complex autoimmune disease with multisystem involvement (82). UC-MSCs treat SLE primarily by suppressing overactivated autoreactive immune cell subsets, reducing autoantibody secretion, and alleviating systemic inflammatory injury.

#### 3.2.1 Preclinical/translational research

UC-MSC-based interventions markedly lowered the frequency of Th1 cells in murine models, with superior efficacy compared with IL-2, a conventional therapeutic agent for SLE (65). In murine lupus models, UC-MSC-EVs alleviated lupus nephritis via multiple protective effects: reducing proteinuria and serum creatinine levels, attenuating renal pathological lesions, lowering the splenic index and serum anti-double-stranded DNA (anti-dsDNA) immunoglobulin G (IgG) titers, and prolonging animal survival. Mechanistically, UC-MSC-EVs restored the homeostasis of the Th1/Th17/Treg axis and suppressed the activation of the IL-6/STAT3 signaling cascade (37).

Modulation of innate immunity and the local inflammatory microenvironment is critical for the therapeutic management of SLE (83). UC-MSCs suppress the differentiation, maturation, and endocytic capacity of monocyte-derived DCs via secreted paracrine factors in a cell-contact-independent manner (45). Similar mechanisms are involved in macrophages. UC-MSC-derived lactate drives M2 macrophage polarization through metabolic reprogramming, accompanied by elevated secretion of IL-6 and TGF-β from DCs (84). An *in vitro* study showed that peripheral blood mononuclear cells (PBMCs) isolated from SLE patients and cultured with UC-MSC-conditioned medium exhibited downregulated CD40 and HLA-DR expression, alongside reduced frequencies of CD19^+^PD-1^+^ and CD19^+^HLA-DR^+^ B-cell subpopulations (51).

#### 3.2.2 Clinical trial progress

The therapeutic application of UC-MSCs in SLE has entered the clinical research stage, and several rigorously designed clinical trials are currently ongoing to evaluate UC-MSC administration in patients diagnosed with moderate-to-severe SLE. Graded cell doses ranging from 1 × 10^6^ cells/kg to 5 × 10^6^ cells/kg are delivered intravenously, with disease activity alterations quantified using the Systemic Lupus Erythematosus Disease Activity Index 2000 (SLEDAI-2K) (66). Zhao et al. devised a randomized, placebo-controlled trial that enrolled 96 patients with refractory lupus nephritis. The renal response rates evaluated at 6 and 12 months were defined as the primary endpoints. Renal biopsy specimens were collected for histological evaluation in participants who achieved a complete clinical response to treatment (67). Additionally, a novel subcutaneous administration strategy using hydrogel-encapsulated UC-MSCs has yielded encouraging interim outcomes in a small case series. Preliminary data demonstrate sustained alleviation of disease activity and favorable steroid-sparing effects, warranting further exploration of this alternative administration route. Specifically, two patients exhibited marked decreases in SLEDAI-2K score (from 20 to 6 and from 10 to 0, respectively), while one patient completely tapered off prednisone; only mild local adverse reactions were documented throughout treatment (68, 69). The therapeutic effects of UC-MSCs may be explained by their ability to mediate the engulfment and clearance of accumulated apoptotic cells. Upon stimulation by apoptotic cells, UC-MSCs markedly upregulate the secretion of PGE_2_, a soluble mediator capable of suppressing T-cell responses (70).

### 3.3 RA

RA is an autoimmune disease characterized by synovial inflammation and joint destruction. Current treatments for RA are accompanied by multiple limitations including the inability to cure the disease; lifelong medication requirements; drug resistance in some patients; and prominent adverse effects such as gastrointestinal injury; elevated infection risk; and hepatic and renal dysfunction (71). UC-MSCs have emerged as a promising candidate therapy to overcome the drawbacks of conventional regimens via regulating abnormal immune responses and repairing damaged joint tissues.

#### 3.3.1 Preclinical/translational research

Multiple studies have compared MSCs from different sources for RA treatment. UC-MSCs home to inflamed synovium while suppressing both innate and adaptive immunity, serving as a candidate cellular therapy for RA (85). In collagen-induced arthritis (CIA) rats, engineered C-C chemokine receptor 2 and IFN-γ-overexpressing UC-MSCs exhibit enhanced joint homing and immunomodulatory capacity alongside decreased lung entrapment relative to native UC-MSCs, as validated by single-cell RNA sequencing (scRNA-seq). These cells facilitate the generation of reparative resting synovial fibroblasts and upregulate cartilage repair-related genes. The secreted semaphorin 3E partially accounts for their anti-inflammatory and chondroprotective properties, identifying a novel paracrine signaling pathway for RA therapy (71).

At the mechanistic level, UC-MSCs exert anti-arthritic effects by remodeling the Treg/Th17 balance. In co-culture systems, UC-MSCs induced a higher Treg/Th17 ratio compared to BM-MSCs. Additionally, UC-MSCs secrete PGE_2_, TGF-β, and IL-10, which suppress Th1 and Th17 differentiation while promoting Treg expansion, thereby inhibiting the inflammatory cascade in joint tissues (86). Abnormal proliferation and apoptosis resistance of fibroblast-like synoviocytes (FLS) are critical drivers of synovial hyperplasia in RA. It has been demonstrated that IL-1β-primed UC-MSCs enhance adhesion to RA-FLS via lymphocyte function-associated antigen-1/intercellular adhesion molecule-1 interaction, and subsequently induce FLS apoptosis through the tumor necrosis factor-related apoptosis-inducing ligand/death receptor 4/death receptor 5 signaling pathway. In the CIA mouse model, IL-1β-preconditioned UC-MSCs markedly alleviate synovial inflammation and joint destruction (72). Despite the local anti-inflammatory activity of IL-1β-primed UC-MSCs, their rapid joint clearance restricts durable immune homeostasis. To achieve long-term Treg-skewed immunosuppression, genetically engineered UC-MSCs secreting mutant IL-2 have been developed for CIA intervention (73).

Growing evidence confirms UC-MSC therapeutic effects are primarily mediated by paracrine secretion. EVs derived from proinflammatory cytokine-primed UC-MSCs contain elevated miR-139-5p, miR-140-5p, and miR-214-5p, which strengthen the immunosuppressive effects of EVs by boosting FOXP3 in healthy human PBMCs. It indicates that UC-MSC-EVs represent a promising cell-free therapy for RA (87). While UC-MSC-EVs exert immunomodulatory effects, glycolytic reprogramming further boosts their potency. Glycolysis-primed UC-MSC-EVs suppressed inflammatory T/B cells and relieved delayed-type hypersensitivity/CIA symptoms via miR-365a-5p-mediated IL-10 upregulation, making them ideal for autoimmune arthritis therapy (88).

#### 3.3.2 Clinical trial progress

Over the past few years, clinical investigations into the application of UC-MSCs in RA have yielded a growing body of robust evidence supporting the safety and therapeutic efficacy of this cell therapy. A prospective I/II study involving 64 active RA patients demonstrated that UC-MSC infusion combined with conventional disease-modifying antirheumatic drugs significantly improved Disease Activity Score 28 (DAS28) and Health Assessment Questionnaire (HAQ) scores, with benefits sustained for up to 3 years and no abnormalities in routine blood tests or liver and kidney function (74). A comprehensive systematic review and meta-analysis of 36 randomized controlled trials (RCTs) (2,076 participants) concluded that MSC therapy, including UC-MSC therapy, has the potential to alleviate joint pain and improve physical function in inflammatory arthritis, without increasing adverse event risks (89). Despite these encouraging findings, all investigations into RA remain within the clinical trial phase, requiring further large-scale randomized controlled trials for long-term efficacy verification.

Insights from BM-MSC studies have also informed RA research. Studies have focused primarily on identifying a safe dose for single-infusion regimens and appropriate delivery routes, typically 1–2 × 10^6^ cells/kg intravenously or 1 × 10^8^ cells via intra-articular injection (90, 91). Repeated infusion has been adopted in only a few studies and has not become the mainstream consensus. Large-scale, well-designed clinical trials are still required to establish the optimal MSC administration protocol for RA.

### 3.4 IBD

IBD mainly consists of ulcerative colitis and Crohn’s disease. Its pathogenesis involves complex interactions among genetic susceptibility, environmental factors, intestinal microbial dysbiosis, and dysregulated immune responses (92). UC-MSCs and their EVs remodel intestinal immunity, repair the mucosa, and alleviate fibrosis via multiple pathways to overcome limitations of conventional IBD therapies, holding great clinical translational potential and serving as a core research focus in regenerative medicine (93).

#### 3.4.1 Preclinical/translational research

The primary mechanism by which UC-MSCs treat IBD is to regulate the homeostasis of immune cells. UC-MSCs are heterogeneous: CD126^hi^ subsets exert anti-inflammatory effects by secreting TGF-β1 to balance Treg/Th1/Th17, whereas CD126^low^ subsets abolish IL-6-induced activation of Th1 and Th17 cells during inflammatory processes. CD126^hi^ UC-MSCs reduce colonic pro-inflammatory cytokines and boost IL-10 in mice with dextran sulfate sodium-induced colitis (75). In line with these cytokine changes, UC-MSCs lowered Disease Activity Index, restored colon length, and alleviated colonic damage in mice with colitis (17). In rat colitis models induced by trinitrobenzenesulfonic acid, intravenously administered UC-MSCs significantly lowered disease activity indices and colon damage scores, alongside mitigation of intestinal inflammatory infiltration. *In vitro* co-culture experiments further confirmed that UC-MSCs secrete tumor necrosis factor-α-stimulated gene 6 (TSG-6). This mediator blocks the Janus kinase 1/STAT1 signaling cascade, curtails M1 macrophage polarization, decreases the expression of pro-inflammatory cytokines, and repairs damaged intestinal tight junctions (76).

Another pivotal therapeutic module of UC-MSCs against IBD lies in the dual remodeling of intestinal physical barrier integrity and gut microecology-mucosal immune homeostasis axis. UC-MSC-EVs reduce mast cell infiltration and intestinal damage by upregulating tight junction proteins Zonula Occludens-1, Occludin, and Claudin-1 and inhibiting the IL-33/suppression of tumorigenicity 2 mast cell activation cascade. Furthermore, UC-MSC-EVs ameliorate IBD-related symptoms and inflammatory responses in murine intestinal epithelial cells via increased global O-GlcNAc glycosylation and suppression of epithelial-mesenchymal transition (94, 95). 16S rRNA sequencing and targeted metabolomics demonstrate that UC-MSCs mitigate colitis and restore T-cell immune homeostasis by enriching short-chain fatty acid-producing bacteria (77).

Nevertheless, in preclinical murine models of IBD, EV-based monotherapy failed to yield significant alleviation of disease pathology. In contrast, a combinatorial regimen comprising intact cells, EVs, and conventional pharmacological agents emerged as a promising therapeutic approach for IBD (96). Importantly, the therapeutic efficacy and bioactivity of EVs as cellular surrogates exhibit considerable heterogeneity across different disease contexts and pathogenic mechanisms, and no broadly accepted framework has yet been established (97).

#### 3.4.2 Clinical trial progress

Three single-arm trials evaluated UC-MSCs for IBD. A 24-week pilot trial of 17 patients with refractory Crohn’s disease who received submucosal and intravenous UC-MSCs showed only mild transient fever. At week 12, the endoscopic response rate was 47%, and the mucosal healing rate was 17.65%; by week 24, all patients achieved full clinical remission and showed reduced inflammatory markers (76). The other cohort of 41 ulcerative colitis participants achieved 73.2% clinical response and 41.5% remission at month 2 (falling to 70.0% and 34.2% at month 6 after 2 dropouts), with improved endoscopic scores, elevated hemoglobin, albumin, and IL-10/IL-17A ratio; age independently predicted efficacy, and no severe adverse events arose (18). Another single-arm trial in 26 patients with ulcerative colitis who received two UC-MSC infusions reported response/remission rates of 80.8%/46.2% at 2 months and 75.0%/37.5% at 6 months. Mechanistically, single-cell RNA sequencing suggested that UC-MSC therapy suppressed T-cell pro-inflammatory features and reduced T-cell crosstalk with B and myeloid cells (17).

One study injected adipose-derived MSCs (AD-MSCs) into IBD fistula tracts, with doses tailored to tract dimensions. Roughly 3 × 10^7^ cells were delivered per centimeter of fistula length for fistulas ≤1 cm wide; the dose doubled for 1–2-cm fistulas. A second injection, if required, used 1.5-fold the initial cell dose (98). While this study reflects a growing interest in local injection strategies, standardized protocols regarding cell dosage and treatment frequency have yet to be established and warrant further investigation. Insights derived from AD-MSC studies may also provide valuable references and guidance for MSC-based research in ulcerative colitis.

## 4 Challenges of UC-MSC therapy

Despite the encouraging results from preclinical and clinical studies, the widespread clinical application of UC-MSCs remains constrained by several critical challenges. Notably, many of these challenges are not unique to UC-MSCs but represent shared hurdles across the MSC field. BM-MSCs, the most extensively studied source, have accumulated the richest repertoire of both successes and setbacks in clinical translation. AD-MSCs and umbilical cord blood-derived MSCs (UCB-MSCs) have also contributed valuable insights into manufacturing scalability and donor variability, respectively. These collective experiences from other MSC sources offer an invaluable repository of lessons learned that can inform and accelerate the clinical development of UC-MSC therapies. In this section, we address key challenges limiting the clinical translation of UC-MSC therapy, including cellular heterogeneity, impaired homing, manufacturing variability, incompletely characterized long-term safety, and clinical trial design. For each bottleneck, we incorporate lessons learned from other MSC sources to illustrate which pitfalls UC-MSCs may intrinsically evade and which obstacles still require proven mitigation strategies.

To offer a structured overview of these translational challenges and, critically, cross-source insights that inform future progress, we summarize the major clinical bottlenecks of UC-MSCs and empirical lessons from better-characterized MSC sources in **Table 2**.

**Table 2:** Cross-source insights for navigating the translational bottlenecks of UC-MSC therapy.

Translational bottlenecks	Lessons from other MSCs	UC-MSC-specific advantages	Applicable mitigation strategies
Cell heterogeneity	BM-MSC: donor age/health cause functional variability, contributing to failed GVHD trials (99, 100). AD-MSC: enzymatic isolation yields heterogeneous subpopulations (101, 102).	Neonatal origin reduces inter-donor variability; Wharton’s jelly provides more homogeneous starting material (103).	Establish donor screening criteria and potency-release assays (BM-MSCs). Define maximum passage number (P8–P10) based on functional stability (104).
Homing deficiency	BM-MSC: <1% infused cells reach target tissues; pulmonary entrapment (105). AD-MSC: local injection effective (Alofisel) but systemic homing poor (106).	UC-MSCs express higher CXCR4/CD44; favorable migration toward inflammatory chemokines (107).	Adopt preconditioning (hypoxia, cytokine priming) validated in BM-MSCs (108). Leverage AD-MSC local delivery for IBD.
Manufacturing inconsistency	AD-MSC: commercial serum-free media and microcarrier platforms established (109). UCB-MSC: extremely low initial cell yield limits expansion consistency (110). BM-MSC: significant lot-to-lot variability in pivotal trials (111).	Abundant Wharton’s jelly-derived starting material; broader proliferation capacity (stable to P10) (103).	Adapt serum-free/xeno-free culture systems. Standardize Wharton’s jelly digestion.
Undefined long-term safety	BM-MSC: >20 years post-marketing safety data available; establishes benchmark (10, 112). AD-MSC: long-term Alofisel follow-up (106).	No tumorigenicity reported; low immunogenicity permits repeated allogeneic dosing without HLA matching (103).	Design prospective long-term registries. Standardize ≥5-year monitoring. Perform genomic stability testing across passages.
Clinical trial design	Most MSC trials failed to stratify patients by inflammatory/immune activation status, masking efficacy signals (lessons from GVHD and IBD trials with BM-MSC/AD-MSC) (113, 114).	UC-MSC trials are mostly Phase II, offering design flexibility (103).	Adopt adaptive/biomarker-driven trial designs to identify responsive patient subsets.

UC-MSC, umbilical cord mesenchymal stromal cell; BM-MSC, bone marrow mesenchymal stromal cell; AD-MSC, adipose-derived mesenchymal stromal cell; UCB-MSC, umbilical cord blood mesenchymal stromal cell; GVHD, graft-versus-host disease; CXCR4, C-X-C motif chemokine receptor 4; IBD, inflammatory bowel disease; HLA, human leukocyte antigen.

### 4.1 Heterogeneity in UC-MSC preparations

Cellular-level heterogeneity constitutes a well-recognized bottleneck across multiple MSC sources. The function of BM-MSCs is highly susceptible to donor age and health status, which largely accounts for the inconsistent outcomes observed in GVHD clinical trials (99, 100). Enzymatic isolation of AD-MSCs inherently generates diverse cell subpopulations with variable biological performance (101, 102). Lessons drawn from these well-studied MSC subtypes can guide the optimization of UC-MSC manufacturing workflows. Owing to their neonatal tissue origin, UC-MSCs derived from Wharton’s jelly exhibit lower inter-donor variability when compared with adult-sourced MSCs (103). Even so, substantial heterogeneity remains widely present within UC-MSC populations.

The umbilical cord consists of several anatomically separated compartments, including Wharton’s jelly, perivascular zone, subamnion, and vascular walls. MSCs harvested from each compartment possess unique phenotypic signatures and functional profiles. Specifically, Wharton’s jelly-derived MSCs hold superior proliferative potential and immunosuppressive potency, whereas perivascular MSCs bear prominent pericyte-like features (115). Intriguingly, functional heterogeneity persists even within MSCs isolated from a single anatomical compartment (i.e., Wharton’s jelly). Single-cell transcriptomic profiling further subdivides Wharton’s jelly-derived MSCs into three discrete subpopulations. Notably, one subpopulation exhibits markedly attenuated immunomodulatory activity and compromised therapeutic performance in murine lupus models (116).

Although UC-MSCs are exempt from the ageing-driven defects seen in BM-MSCs, primary cells collected from distinct donors carry intrinsic genetic and epigenetic discrepancies that trigger inter-donor functional heterogeneity and hinder clinical translation. More than 10 independent UC-MSC lines from separate donors expressed classic MSC surface markers yet displayed stark functional gaps, especially in Treg induction and T-lymphocyte suppression (117). A 2025 study in Stem Cell Research & Therapy systematically evaluated UC-MSCs from 10 healthy donors, classifying them into high, medium, and low immunomodulatory profiles based on T-cell proliferation inhibition. Notably, even under pro-inflammatory stimulation (IFN-γ and TNF-α), IDO expression varied markedly: 7 donors exceeded 60% expression, while 3 donors fell below 30%, with one donor showing ≤2%. This finding underscores that donor-dependent functional disparities persist despite standardized culture conditions (118).

Donor sex is a critical intrinsic determinant of molecular heterogeneity in UC-MSCs. UC-MSCs derived from male donors show significantly higher octamer-binding transcription factor 4 (OCT4) and DNA methyltransferase 1 (DNMT1) transcription than in female-derived cells (119). Twin-cohort transcriptomic comparisons further uncovered sex-linked functional gaps: male UC-MSCs show faster proliferation, strengthened adipogenic differentiation, up-regulated pluripotency genes (Nanog homeobox, OCT4, telomerase reverse transcriptase, SRY-box transcription factor 2), and elevated pro-inflammatory cytokine secretion upon lipopolysaccharide (LPS) stimulation (120). Such sex-dependent traits carry clear implications for UC-MSC immunotherapy against inflammatory disorders.

Isolation and cultivation protocols markedly determine the quality of UC-MSCs. The basic fibroblast growth factor (bFGF)-aided enzymatic digestion yields superior cell viability and proliferation compared with mechanical shearing; bFGF remodels metabolic pathways and regulates adhesion, morphogenesis, and immunomodulation-related proteins (121). Culture-medium composition is equally critical. A study that enrolled 90 donors verified that culture medium supplemented with human platelet lysate outperforms fetal bovine serum (FBS) for UC-MSC expansion. Optimized standardized workflows achieve 98.9% cellular purity and above 97% cell viability, and ensure reproducibility across different donor batches. Replacing xenogeneic FBS with human platelet lysate enhances batch consistency and biological safety. Animal-free, good manufacturing practice (GMP)-compliant operating procedures are mandatory to produce clinical-grade UC-MSC preparations (122).

For the industrialization of UC-MSC therapies, improving cell homogeneity is essential to realize standardized cell production and facilitate consistent clinical outcome analysis. One landmark study developed a mixed-donor cell pooling strategy to mitigate inter-donor divergence in immunomodulatory activity; meanwhile, multiple priming approaches have been validated to support standardized manufacturing of cell-based drugs (123). Based on their ability to suppress T-cell proliferation *in vitro*, pooling UC-MSCs isolated from high-function and low-function donors at a 1:2 proportion will effectively elevate the overall immunoregulatory capacity of inferior batches (118).

While intrinsic variability is a significant hurdle for product consistency, it also presents a strategic opportunity. By adopting cellular heterogeneity as a screening metric, we may select UC-MSC strains whose functional profiles are optimally matched to specific inflammatory pathologies. This approach shifts the paradigm from merely minimizing heterogeneity to actively leveraging it, establishing a pathogenesis-driven evaluation framework that may surpass conventional therapeutic benchmarks.

### 4.2 Homing and biodistribution

Systemic administration of UC-MSCs results in extensive pulmonary entrapment due to the first-pass effect, with over 95% of infused cells sequestered in the lungs and subsequently cleared within 72 hours in both immunocompetent and immunocompromised hosts. This pulmonary entrapment initiates an acute inflammatory phase immediately post-injection, which typically transitions into a resolution phase within 24 hours (124, 125). While this transient pulmonary response is therapeutically advantageous for treating local inflammatory conditions such as acute respiratory distress syndrome or COVID-19, it concurrently limits the bioavailability of UC-MSCs reaching distal target organs, thereby constraining their systemic therapeutic efficacy.

Multiple proven approaches from BM-MSC research are adaptable to boost UC-MSC homing performance. Hypoxic preconditioning and cytokine priming, which have been validated for BM-MSCs, can improve the migratory phenotype of UC-MSCs (108). Although the Phase III trial of AD-MSC-based Alofisel missed its primary endpoint, its local-delivery strategy offers a valuable model for UC-MSC-based local injection in IBD (106). Additional optimized delivery tactics for UC-MSCs cover intralesional injection, cell-surface modification, and temporary pulmonary vasodilation (126).

The homing ability of UC-MSCs is mainly regulated by the expression of chemokine receptors and the abundance of their soluble ligands. Recent studies have identified the CXCL10-C-X-C motif chemokine receptor 3 axis and IL4I1 secretion as core regulatory pathways. Targeting these pathways effectively enhances the therapeutic effects of UC-MSCs in the treatment of lupus nephritis (127).

Despite these delivery optimization strategies, the intrinsic pharmacokinetic complexity of UC-MSCs remains a challenge for conventional evaluation frameworks. As viable cell therapeutics, UC-MSCs possess intrinsic proliferative and migratory features. Their *in vivo* circulation and clearance behaviors differ drastically from conventional small-molecule drugs, making standard pharmacokinetic models inapplicable. Current analytical tools have limited our understanding of the dynamic biodistribution, retention at the lesion site, and fate of infused UC-MSCs in patients. Distinct from other cell therapies, UC-MSCs act via a “hit-and-run” mechanism: they rapidly home to injured tissues, secrete paracrine mediators (cytokines, growth factors, hormones, miRNAs) to remodel local microenvironments, and are subsequently cleared. Even with transient tissue residence, they generate sustained therapeutic effects (128).

### 4.3 Standardization and quality control

Manufacturing-related inconsistency represents a major obstacle to the scalable clinical translation of MSC products. Notably, other MSC sources have accumulated considerable experience in process development. For instance, AD-MSCs already benefit from commercially available serum-free media and microcarrier-based expansion platforms (109). These strategies may offer valuable references for the scalable production of UC-MSCs. However, UCB-MSCs suffer from extremely low initial cell yields, which hinder stable cell expansion, and BM-MSCs exhibit prominent batch-to-batch variation throughout pivotal clinical trials (110, 111). These issues are ultimately rooted in the intrinsic limitations of their respective tissue origins. Compared with these adult tissue-derived MSCs, UC-MSCs take advantage of abundant Wharton’s jelly starting materials and superior proliferative capacity that remains stable up to passage 10 (P10) (103), circumventing the sourcing constraints from the outset and positioning them as uniquely promising candidates for scalable manufacturing.

In terms of quality control system construction, previous studies have established a full-process framework covering donor screening (infectious disease testing plus re-examination after 3 months), viral testing of raw materials, a two-tier system of master and working cell banks, and release assays including sterility, mycoplasma testing, and endotoxin measurement (<0.5 EU/mL). UC-MSCs manufactured under this system produced no serious adverse events during a 1-year follow-up of 225 patients (129). Furthermore, a full GMP-compliant quality control system covers three core sections: cellular characterization, biosafety testing, and functional potency evaluation. Qualified cells show ≥95% positivity for standard positive markers CD73, CD90, and CD105, and <2% expression of negative markers CD34 and HLA-DR. Pre-cryopreservation cell viability must exceed 90% to guarantee cell quality. Biosafety assays include karyotyping, micronucleus test, and soft agar colony formation assay; T-cell proliferation inhibition assays are adopted to verify cellular immunosuppressive potency (130).

Current release criteria, which mainly depend on surface marker phenotyping and trilineage differentiation potential, cannot adequately predict the therapeutic efficacy of UC-MSCs. Therefore, it is imperative to incorporate functional assays, such as T-cell suppression tests or IDO activity measurements, to address this gap (118). The recently updated International Society for Cellular Therapy (ISCT) criteria for MSC characterization have incorporated tissue-specific marker assays to address the inherent cellular heterogeneity of umbilical cord tissue, thereby enhancing phenotypic characterization and refining purity assessment of clinical-grade UC-MSC products (131). On the other hand, cellular passage represents a decisive variable governing UC-MSC quality and therapeutic performance. Early-to-mid passages (P3–P5) strike an optimal equilibrium between proliferative expansion capacity and immunoregulatory functionality.

### 4.4 Long-term safety

Long-term safety remains a key translational bottleneck for UC-MSCs. Lessons drawn from other MSC sources indicate that BM-MSCs have served as a safety benchmark, with more than 20 years of post-marketing data (10, 112), and Alofisel (AD-MSCs) has demonstrated well-documented long-term safety profiles (10, 106, 112). For UC-MSCs, current evidence suggests that these cells exhibit no tumorigenicity and extremely low immunogenicity, permitting repeated allogeneic infusions in the absence of HLA matching (103). This observation is further supported by preclinical studies, in which even ultra-high doses of UC-MSCs did not induce tumor formation in immunodeficient mice over a 26-week observation period (132).

Existing clinical evidence generally supports a favorable safety profile; however, most studies are based on relatively small cohorts, and larger, well-controlled trials are still needed to substantiate these findings. The longest follow-up data come from a study involving patients with multiple sclerosis and neuromyelitis optica, which suggested that combined intravenous and intrathecal administration of UC-MSCs is safe and feasible. None of the serious adverse events, such as tumor formation and peripheral organ and tissue disorders, were observed throughout the 10-year follow-up (133). Recently, a Phase I trial enrolling patients with ulcerative colitis extended the monitoring duration to 24 months. No adverse events were identified at the 12- and 24-month follow-up assessments, indicative of sustained safety (134). Supporting evidence also comes from a 5-year follow-up survey in healthy volunteers, which showed that all participants remained in good health with no side effects or major health issues; tumor biomarkers stayed within normal ranges, key organ functions remained stable, and a sustained dose-dependent anti-inflammatory effect was observed in the high-dose group (135).

Collectively, the literature supports a reassuring long-term safety profile for UC-MSC therapy in immune-related diseases, though all investigators concur that large-scale clinical validation remains necessary.

### 4.5 Clinical trial design

The biological challenges of UC-MSC therapy discussed above ultimately converge at the clinical-trial level. Even a well-characterized, optimally manufactured UC-MSC product will fail to demonstrate efficacy if the trial design is not powered to detect biologically meaningful signals within the appropriate patient population. A systematic analysis of Phase I and III MSC trials concluded that while numerous pilot and Phase I/II studies have demonstrated safety and promising efficacy signals, the majority of Phase III studies have yielded negative results upon progression to pivotal testing (136). Among the identified challenges, inadequate patient stratification, heterogeneous outcome definitions across study sites, and a lack of predictive-biomarker monitoring were cited as recurring design flaws that attenuate observed treatment effects (113, 114). These lessons are directly transferable to UC-MSC development, which remains predominantly at the Phase I/II stage and therefore offers an opportunity to incorporate improved trial-design strategies from the outset.

A recent UC-MSC trial has underscored the critical importance of patient stratification for unlocking MSC therapeutic potential in the treatment of aGVHD. This randomized, double-blind Phase I/II trial enrolled 22 patients with grade II–IV aGVHD and found no significant differences between the UC-MSC and placebo groups in the day-28 overall response rate or 12-month survival. However, subgroup analysis further revealed that among patients with severe grade III–IV aGVHD who achieved an early response, those treated with UC-MSCs exhibited significantly better 12-month survival compared with placebo recipients (100% vs. 50%) (137). Notably, this subgroup-specific benefit is not exclusive to UC-MSCs. Baseline immune profiling identified CD4^+^ terminally differentiated effector memory T cell (TEMRA) >35% and CD8^+^ TEMRA >70% as predictive biomarkers for BM-MSC survival benefits, which were masked in unstratified cohort analysis (99). The failure to stratify patients by inflammatory and immune activation status has been repeatedly cited as a key reason why MSC trials, across BM-MSC, AD-MSC, and UCB-MSC candidates, have failed to confirm Phase II signals in pivotal studies (138).

Beyond patient selection, UC-MSC trial design must also address the dosing regimen. The optimal dosing schedule, including cell number, frequency, and route of administration, remains poorly defined, yet these parameters critically influence homing, biodistribution, and therapeutic durability. Emerging UC-MSC trials are beginning to incorporate repeated dosing at predefined intervals; for example, a Phase I/II trial for diabetic peripheral neuropathy administers three intramuscular UC-MSC injections on day 0, day 7, and day 28, with extended safety follow-up through week 104 (139). This design effectively reconciles biological plausibility with regulatory acceptability, as sustained immunomodulation requires repeated exposure while clearly defined safety monitoring windows satisfy regulatory expectations.

Building upon cumulative experience from earlier MSC studies and emerging UC-MSC evidence, researchers have identified several actionable strategies to optimize future trial design. Biomarker-driven patient enrichment should be implemented prospectively rather than through post-hoc analysis. The recently proposed “Decode-Map-Design” paradigm for UC-MSCs integrates single-cell and spatial omics to define the functional states of MSC products, enabling mechanism-guided patient stratification by aligning cell-product properties with disease pathogenesis (103). This strategy converts patient heterogeneity from a major confounding factor into a targetable therapeutic opportunity. Complementing these design-level innovations, the ISCT has emphasized that standardized reporting is essential for MSC trials in autoimmune disorders, enabling accurate data interpretation and robust meta-analyses from comparable datasets (131). Without such standardization, even well-conducted UC-MSC trials will remain challenging to compare, ultimately hindering evidence accumulation.

The UC-MSC field is developing rapidly. Hard-won lessons from previous work can inform the design of smarter, biomarker-driven, adaptive trials that align with the biological complexity of both the cell product and its target diseases. Critically, Phase II trial designs should prioritize moderate-dose, fractionated-administration regimens and justify route selection based on disease pathophysiology, rather than relying on empirical or convenience-based choices.

## 5 Optimization of UC-MSC culture and expansion strategies

The path to large-scale clinical translation of UC-MSCs in treating various immune disorders is fraught with challenges, as outlined above. Many of these obstacles are attributable to the *in vitro* culture microenvironment, yet traditional culture systems fail to recapitulate the physiological niche of UC-MSCs (140). Consequently, a growing body of research has focused on multiple culture optimization strategies and genetic engineering aimed at preserving stemness, delaying senescence, and enhancing immunoregulatory function. This section systematically reviews the mainstream optimization approaches, including cytokine priming, hypoxic preconditioning, three-dimensional (3D) spheroid culture, and genetic engineering (**Figure 3**).

**Figure 3:**
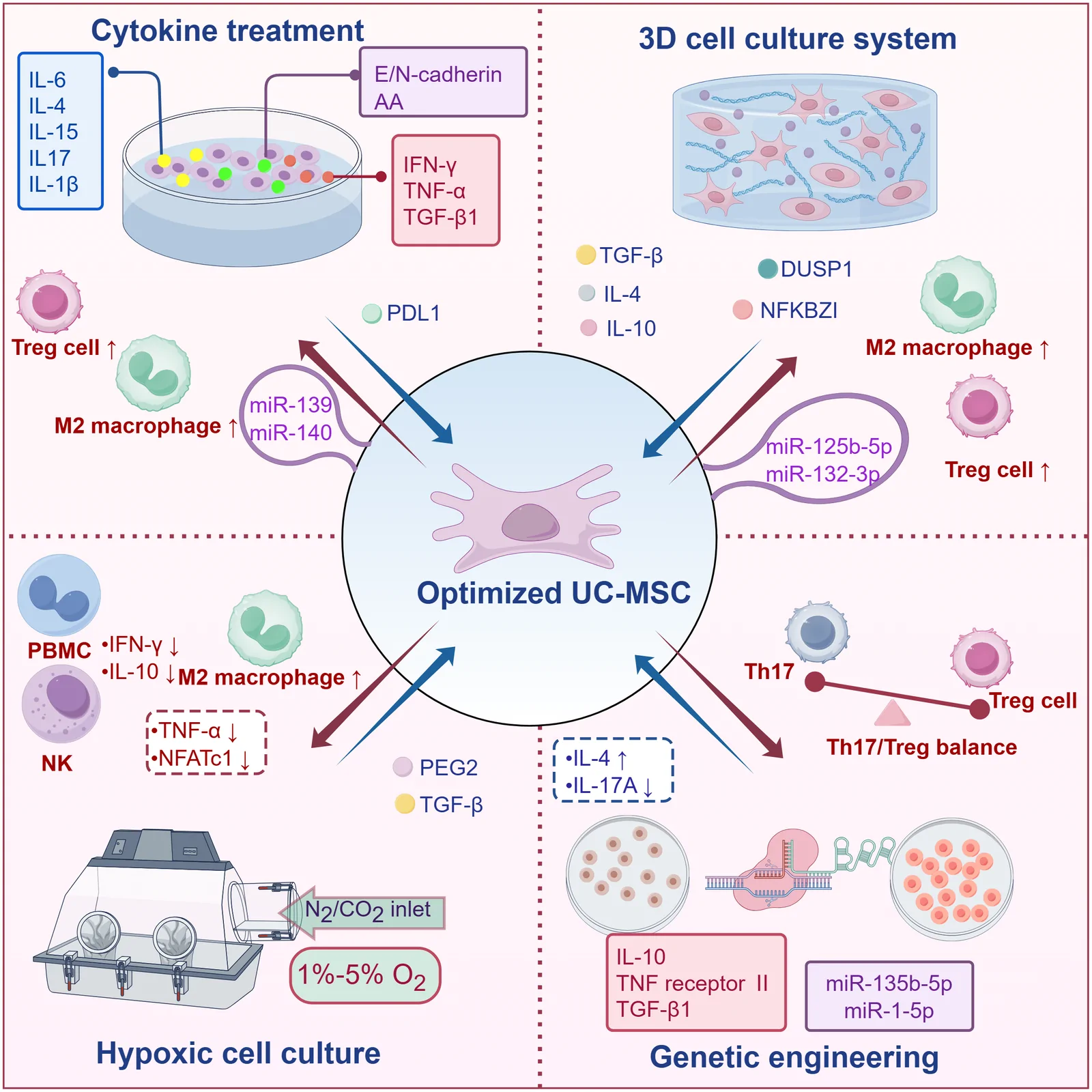
Optimization of UC-MSC culture and expansion strategies. The immunomodulatory efficacy of UC-MSCs can be significantly enhanced through various preconditioning and engineering strategies. Cytokine priming with TNF-α, TGF-β1, and IFN-γ, particularly in combination with E-/N-cadherin and all-trans retinoic acid (AA), promotes Treg induction and M2 macrophage polarization. Three-dimensional (3D) culture exerts similar effects on Treg and M2 polarization, while also upregulating the production of immunomodulatory molecules such as TGF-β, IL-4, IL-10, DUSP1, and NFKBIZ. Hypoxic preconditioning (1%–5% O_2_) enhances M2 polarization and suppresses cytokine secretion from PBMCs and NK cells, with notable reduction in IFN-γ and IL-10. Genetic modification, typically involving overexpression of immunomodulatory cytokines or exosome-related genes, allows fine-tuning of the Th17/Treg balance and overall cytokine profiles, as exemplified by upregulation of IL-4 and downregulation of IL-17A. Collectively, these approaches provide versatile tools for optimizing UC-MSC-based immunotherapies.

### 5.1 Cytokine preconditioning

Exposing UC-MSCs to pro-inflammatory cytokines, particularly IFN-γ alone or in combination with TNF-α and IL-1β, enhances their immunomodulatory potency. This “licensing” process upregulates IDO, PD-L1, and MHC-I expression (141). The single-cell transcriptomes of UC-MSCs after priming with six cytokines: IFN-γ, TNF-α, IL-4, IL-6, IL-15, and IL-17 demonstrated that cytokine priming minimized the heterogeneity of the UC-MSC transcriptome, comprehensively activated signaling pathways targeting T cells and macrophages, while the expression of UC-MSC surface markers exhibited only slight changes (142). More results suggest that the co-induction of IFN-γ and TNF-α upregulates PD-L1 expression in UC-MSCs, accompanied by a substantial increase in miR-139-5p and miR-140-5p cargo packaged into UC-MSC-EVs. These EVs expanded FOXP3^+^ Tregs, targeting SLE, RA, and Sjögren’s syndrome (143, 144). IFN-γ/TGF-β1-primed UC-MSCs significantly upregulated CD25^+^ expression on T cells, while suppressing IL-6 secretion, without affecting T-cell proliferation rate. This may point to potentially stronger antiviral effects, while alleviating the viral infection-induced cytokine storm (145). In the CIA mouse model, RA symptoms and inflammation were significantly improved with IL-1β-stimulated UC-MSC administration, highlighting the unique therapeutic advantages of IL-1β-primed UC-MSCs for RA intervention (72).

Beyond single-agent or combined cytokine priming, the incorporation of other functional molecules also plays a vital role in optimizing UC-MSC therapeutic efficacy. IFN-γ and ascorbic acid (AA) co-preconditioned UC-MSCs suppress LPS-triggered cytokine storm by reducing mRNA and protein levels of IL-6, IL-1β, and TNF-α relative to control groups. Such dual priming also facilitates angiogenesis in yolk sac membrane assays and reshapes the secretome with unique growth factor and cytokine signatures. Collectively, dual IFN-γ and AA preconditioning strengthens UC-MSC therapeutic potency, making these primed cells promising candidates for cell therapy against COVID-19 and various inflammatory disorders (146). IL-6 and E/N-cadherin modification-primed 3D UC-MSC aggregates exhibited enhanced adhesion, anti-apoptosis, immunosuppression, and angiogenesis during the treatment of systemic sclerosis. Moreover, these aggregates exhibited improved immunoregulatory effects on CD4^+^ and CD8^+^ T cells *in vitro* and *in vivo* (147).

Emerging studies have verified the therapeutic potential of cytokine preconditioning for UC-MSCs, yet this strategy is still exploratory. Preconditioning induces UC-MSC transcriptional reprogramming, reduces stemness subpopulations, and induces cells to polarize toward an immunoregulatory phenotype, accompanied by a loss of intrinsic tissue-repair functions and decreased multilineage differentiation potential, including chondrogenic differentiation (142, 148). It also induces apoptosis and senescence in UC-MSCs, which suggests that infused cells exhibit accelerated *in vivo* clearance, truncated functional duration, and impaired long-term therapeutic effects (34). Moreover, existing data are largely derived from *in vitro* assays, while large-animal and long-term *in vivo* studies remain scarce, leaving clinical efficacy and safety largely undefined.

### 5.2 Hypoxic preconditioning

UC-MSCs natively reside within a niche characterized by 1%–5% O_2_ (physiological hypoxia; median approximately 5% O_2_), whereas the conventional normoxic culture condition of 21% O_2_ imposes a supraphysiological hyperoxic stress. Compared with normoxic culture, physioxia (1%–5% O_2_) better recapitulates the endogenous microenvironment, markedly boosting the secretion of immunosuppressive factors including PGE_2_, TGF-β, and vascular endothelial growth factor, reinforcing stemness and proliferation, and facilitating post-transplant survival and homing capacity (149). Taken together, these favorable properties demonstrate superior translational potential and clinical applicability of the protocol. Hypoxic UC-MSCs (3% O_2_) significantly decreased the IFN-γ levels and elevated the IL-10 levels in PBMCs from patients with SLE compared with those in the healthy group (150). In a rat model of peri-implantitis, infusion of hypoxia-preconditioned UC-MSCs (5% O_2_) markedly attenuated both inflammatory responses and osteoclastogenesis. Notably, this was associated with a significant downregulation of TNF-α and nuclear factor of activated T cells 1 and a concurrent upregulation of osteocalcin and collagen type I alpha 1, when compared to the control group (151).

Most current investigations merely perform short-term hypoxic priming of UC-MSCs for 24–48 h. Nevertheless, a distinctive investigation established a persistent hypoxic culture system with 1% O_2_, in which UC-MSCs were continuously incubated from passage 0 up to passage 6 as the experimental group throughout the entire experimental workflow. Subsequent experiments indicate that consecutive hypoxic cultures could not only promote proliferation and quality of UC-MSCs but also boost the therapeutic potency of their secretome to alleviate pulmonary inflammation via facilitating efferocytosis and driving anti-inflammatory macrophage polarization (152).

Two classic priming strategies, inflammatory cytokine stimulation and hypoxic preconditioning, have been shown to synergistically amplify the immunomodulatory capacity of UC-MSCs when applied in combination (153). A classic study demonstrated that combined pretreatment with hypoxia (2% O_2_) and inflammatory factors (IFN-γ, TNF-α, and IL-1β) elongated UC-MSC morphology without affecting cell viability, proliferation, or size. Pretreatment significantly downregulated coagulation-related tissue factors without altering other surface markers, while maintaining mitochondrial function and structural integrity. Notably, although pretreatment promoted apoptosis and senescence in UC-MSCs, it upregulated the expression of immunoregulatory genes and proteins. When NK cells were co-cultured with pretreated UC-MSCs, their cytotoxicity was inhibited to varying degrees (34).

A critical unresolved issue remains for hypoxia-preconditioned UC-MSCs, as their efficacy depends on preconditioning variables. Hypoxic duration and oxygen concentration strongly determine outcomes, and inappropriate conditioning may not merely reduce effectiveness but actively convert the cells into a harmful phenotype (154). Of the data on hypoxic UC-MSCs, 95% come from *in vitro* and small-animal pilot studies; long-term safety cohorts in large-animal studies and complete human clinical trial data are lacking. In addition, hypoxia-preconditioned UC-MSCs possess acceptable biosafety as assessed by routine testing, yet high-dose infusion is associated with venous thrombosis and disseminated embolism in multiple organs, resulting in animal death (155). Therefore, thrombogenic risk should be fully considered in future clinical translation and application of hypoxia-primed UC-MSCs.

### 5.3 3D culture and spheroid formation

UC-MSCs are conventionally cultured in static 2D monolayers attached to plastic flasks. Lacking physiological 3D cell–cell contacts, this system fails to recapitulate *in vivo* niches and yields cells with restricted therapeutic potency. Accordingly, 3D culture models that mimic the native complex microenvironment have attracted growing research interest (156). Compared with conventional 2D culture, the 3D microcarrier–bioreactor system substantially boosted the proliferation, viability, stemness, and immunomodulatory competence of UC-MSCs, as evidenced by elevated expression of IL-4, IL-10, and TGF-β. Meanwhile, this culture system preserved genomic stability, canonical surface marker profiles, non-tumorigenic potential, and multilineage differentiation capacity (157).

The recombinant fusion protein hE-cadherin-Fc, consisting of the human E-cadherin extracellular domain and the IgG Fc segment, acts as a cell-adhesive biomimetic substrate to support UC-MSC expansion and effectively facilitates cell adhesion and proliferation. UC-MSCs expanded on this system maintained CD105 positivity and exhibited upregulated E-cadherin and β-catenin expression (147). Functionally, conditioned supernatants from 3D-cultured UC-MSCs blocked IL-17A-triggered NF-κB activation in keratinocytes. *In vivo*, 3D-cultured UC-MSCs markedly attenuated psoriatic skin inflammation in mouse models by reducing immune cell infiltration and suppressing IL-17-driven inflammatory cascades (157). In a different approach, UC-MSCs grown in 3D spheroid or microcarrier systems exhibit enhanced immunomodulatory properties due to increased cell-cell contact and upregulated anti-inflammatory genes. 3D spheroids were constructed by suspension stirring and cultured in serum-free conditions for 4–7 days to obtain 3D-conditioned medium. In rat adjuvant-induced arthritis models, 3D-conditioned medium elevated secretion of IL-10, leukemia inhibitory factor, and platelet-derived growth factor-BB, alleviating paw swelling, reducing arthritis scores, and suppressing synovial inflammation and bone erosion, with superior efficacy to 2D-derived medium (158).

3D-cultured UC-MSCs demonstrate broad therapeutic advantages over 2D cultures, driven by enhanced immunomodulation and angiogenesis (159). *In vivo* results demonstrated the superior therapeutic efficacy of 3D-cultured UC-MSC derivatives across multiple diabetic and autoimmune models. In a rat type 1 diabetes model, 3D-conditioned medium effectively promoted Treg expansion and preserved pancreatic β-cell mass. In mice with diabetic wounds, 3D-cultured UC-MSCs accelerated cutaneous repair by enhancing angiogenesis and suppressing inflammation. In mice with vitiligo, EVs from 3D-cultured UC-MSCs, enriched with miR-132-3p and miR-125b-5p, alleviated depigmentation by potentiating Treg-mediated immune tolerance and inhibiting oxidative stress-induced melanocyte apoptosis (159–161). Additional studies confirmed that 3D culture preserved the stemness of UC-MSCs and boosted immunomodulatory efficacy in colitis and type 1 diabetes models (162). Mechanistically, 3D spheroids increased EV secretion by approximately 6.7-fold compared with 2D cultures, with TNF-α and IFN-γ priming further enhancing production to 9.4-fold. Collectively, 3D culture combined with inflammatory priming in defined media potently augments the regenerative functions of UC-MSCs, including angiogenesis, wound healing, anti-inflammation, anti-apoptosis, and anti-fibrosis (163).

Current 3D culture systems still present notable limitations. Stable cultivation relies on sophisticated procedures, including scaffold fabrication, precise cell seeding, and stringent process control. Given the dynamic remodeling of 3D matrices, biomaterials are required to recapitulate the mechanical properties and degradability of target tissues, necessitating comprehensive physicochemical characterization to ensure reproducible outcomes (164). Moreover, the preparation of high-quality single-cell suspensions from umbilical cord tissue remains a significant technical hurdle, as mechanical and enzymatic dissociation processes frequently compromise cell viability and structural integrity, thereby reducing the yield and functional quality of the starting cell population (165). Furthermore, the high cost of 3D culture reagents remains an economic barrier to the scale-up of 3D culture (166).

### 5.4 Genetic engineering

Native UC-MSCs encounter multiple drawbacks: low *in vivo* viability, insufficient tissue-homing ability, moderate immunoregulatory potency, progressive stemness loss upon serial passaging, and inherent thrombotic risk. Precise genetic engineering enables targeted regulation of core genes to prolong cell survival, augment lesion-specific homing, strengthen anti-inflammatory capacity and differentiation potential, improve the safety profile of cell transplantation, and endow UC-MSCs with distinctive therapeutic functions. Collectively, these modifications greatly enhance the therapeutic potency of UC-MSCs in preclinical and translational investigations covering arthritis, repair of tissue injury, transplant rejection, malignant tumors, and fibrotic disorders (167).

Cytokine overexpression represents the primary strategy for genetic engineering of UC-MSCs. Lentivirus-mediated overexpression of IL-10 in UC-MSCs markedly promotes intestinal hyperplasia and angiogenesis, thereby accelerating colonic tissue repair in mouse colitis models (168). Modification with TNF receptor 2 protects UC-MSCs against TNF-α-induced apoptosis and autophagy and effectively alleviates arthritis in mice by promoting migration to affected areas, protecting articular cartilage from destruction, and modulating immune balance (169).

Another strategy for UC-MSC genetic engineering involves miRNA-mediated modification. UC-MSCs overexpressing miR-125b-5p downregulate Th17-related cytokine expression, correct the Th17/Treg immune imbalance, and regulate the expression of IL-17 and IL-4. Additionally, this modification reduces immune complex deposition and inflammatory cell infiltration in the lung and kidney tissues of mouse models of lupus (170). In mice with lupus, miR-1-5p-overexpressing UC-MSCs attenuated liver and intestinal damage through suppression of the JAK2/STAT3 pathway and IL-18 expression (171).

Clustered regularly interspaced short palindromic repeats (CRISPR)-Cas9-mediated gene editing facilitates the generation of universal off-the-shelf UC-MSCs with improved resistance to allogeneic immune rejection while maintaining intact immunomodulatory functions, rendering these cells highly promising candidates for clinical translation. A recent study demonstrated that CRISPR-Cas9-mediated β-2-microglobulin knockout combined with adeno-associated virus-driven knock-in of the β-2-microglobulin-HLA-G fusion gene enables the generation of engineered UC-MSCs that not only resist T- and NK-cell-mediated lysis but also preserve full immunomodulatory activity. This strategy provides a viable route to construct off-the-shelf universal donor cells with enhanced *in vivo* persistence and improved clinical translatability (172).

Although genetically edited UC-MSCs exhibit therapeutic promise, critical hurdles persist: off-target mutations, long-term *in vivo* safety, and scalable production under GMP conditions. Few labs have replicated these results, requiring cross-model verification. Existing studies confirm that gene editing boosts UC-MSC reparative ability, yet major translational and safety barriers must be resolved before clinical application (173). Furthermore, owing to inherent limitations of gene-editing technologies, non-integrating vectors offer only transient expression, while the long-term *in vivo* risks associated with integrating vectors remain unknown. These uncertainties render clinical translation particularly challenging; substantial foundational research is required.

## 6 Discussion

UC-MSCs have emerged as a promising immunomodulatory platform for immune-mediated disorders, underpinned by the robust pleiotropic effects summarized above. As one of the MSC types with the broadest range of applications, UC-MSCs exert potent immunomodulatory effects across innate and adaptive immune compartments by balancing the Th17/Treg axis, promoting the differentiation of Breg cells, facilitating macrophage polarization from M1 to M2, inhibiting DC maturation, and suppressing the proliferation and differentiation of NK cells, plasma cells, and neutrophils. Cumulative preclinical data across a spectrum of immune-mediated inflammatory disorders consistently demonstrate that UC-MSC administration restores disrupted immune tolerance and alleviates inflammatory tissue injury. Encouragingly, a growing body of early-phase clinical trials has translated these experimental observations into human studies, generating preliminary evidence supporting the safety and therapeutic potential of UC-MSCs in multiple immune-mediated conditions.

Nevertheless, existing clinical studies are limited by multiple interlinked barriers. First, a lack of unified manufacturing protocols leads to variable cell quality between laboratories and manufacturers, with differences in isolation procedures, culture media, passage number, cryopreservation conditions, and cell dose contributing to inconsistent functional properties of infused UC-MSCs. Second, there exists no consensus on optimized treatment regimens, including administration timing, route of delivery, cell dosage, and treatment frequency; these variables strongly influence cell engraftment, survival within inflammatory microenvironments, and downstream immune regulation. Furthermore, many preclinical investigations rely on simplified animal models that poorly recapitulate the complexity of chronic human immune diseases, limiting the translational value of *in vivo* experimental results. Conflicting findings persist regarding the long-term safety profile of repeated UC-MSC infusion, the risk of off-target immune modulation, and the durability of therapeutic responses. Importantly, most existing mechanistic studies characterize UC-MSC function under static *in vitro* conditions, while how the inflammatory milieu dynamically reshapes UC-MSC phenotype after systemic delivery remains incompletely understood. Collectively, current literature establishes a solid mechanistic foundation to justify continued investigation of UC-MSCs as immunoregulatory therapeutics, yet substantial gaps separate descriptive mechanistic findings from consistent, reproducible clinical benefit.

In response to the translational limitations, diverse engineering and priming strategies have been developed to augment the potency, stability, and environmental adaptability of UC-MSCs, offering actionable directions to overcome current bottlenecks. Inflammatory preconditioning, hypoxia pretreatment, 3D culture systems, and genetic modification all aim to strengthen the secretory profile and immunoregulatory activity of UC-MSCs, improving their resistance to hostile inflammatory tissue microenvironments. Each strategy carries unique advantages and inherent limitations: priming approaches are relatively straightforward to integrate into existing cell production workflows, whereas genetic engineering confers persistent functional enhancement but introduces additional safety and regulatory considerations. Moving forward, several priorities should guide subsequent research efforts. Further work is required to establish standardized release criteria to define functionally competent UC-MSC products based on predictive immunomodulatory biomarkers. Head-to-head comparisons of optimized priming protocols will help identify cost-effective, clinically scalable approaches to enhance therapeutic consistency. Additionally, future clinical trials ought to incorporate stratified study designs to clarify how patient characteristics, disease stage, and inflammatory status influence UC-MSC treatment responses. Ultimately, refining UC-MSC preparation protocols, clarifying dose-response relationships, and aligning mechanistic research with clinically relevant endpoints will facilitate the transition of UC-MSC therapy from experimental immunology toward standardized precision treatment for immune-mediated diseases.
